# Restriction of SARS-CoV-2 replication by targeting programmed −1 ribosomal frameshifting

**DOI:** 10.1073/pnas.2023051118

**Published:** 2021-06-14

**Authors:** Yu Sun, Laura Abriola, Rachel O. Niederer, Savannah F. Pedersen, Mia M. Alfajaro, Valter Silva Monteiro, Craig B. Wilen, Ya-Chi Ho, Wendy V. Gilbert, Yulia V. Surovtseva, Brett D. Lindenbach, Junjie U. Guo

**Affiliations:** ^a^Department of Neuroscience, Yale University School of Medicine, New Haven, CT 06520;; ^b^Yale Center for Molecular Discovery, Yale University, West Haven, CT 06516;; ^c^Department of Molecular Biophysics and Biochemistry, Yale University School of Medicine, New Haven, CT 06520;; ^d^Department of Microbial Pathogenesis, Yale University School of Medicine, New Haven, CT 06520;; ^e^Department of Laboratory Medicine, Yale University School of Medicine, New Haven, CT 06520;; ^f^Department of Immunobiology, Yale University School of Medicine, New Haven, CT 06520

**Keywords:** translation, ribosomal frameshifting, RNA pseudoknot, coronavirus, merafloxacin

## Abstract

Translation of open reading frame 1b (ORF1b) in severe acute respiratory syndrome coronavirus 2 (SARS-CoV-2) requires a programmed −1 ribosomal frameshift (−1 PRF) promoted by an RNA pseudoknot. The extent to which SARS-CoV-2 replication may be sensitive to changes in −1 PRF efficiency is currently unknown. Through an unbiased, reporter-based high-throughput compound screen, we identified merafloxacin, a fluoroquinolone antibacterial, as a −1 PRF inhibitor for SARS-CoV-2. Frameshift inhibition by merafloxacin is robust to mutations within the pseudoknot region and is similarly effective on −1 PRF of other betacoronaviruses. Consistent with the essential role of −1 PRF in viral gene expression, merafloxacin impedes SARS-CoV-2 replication in Vero E6 cells, thereby providing proof-of-principle for targeting −1 PRF as a plausible and effective antiviral strategy for SARS-CoV-2 and other coronaviruses.

## Significance

A large variety of RNA viruses, including the novel coronavirus SARS-CoV-2, contain specific RNA structures that promote programmed ribosomal frameshifting (PRF) to regulate viral gene expression. From a high-throughput compound screen, we identified a PRF inhibitor for SARS-CoV-2 and found that it substantially impeded viral replication in cultured cells. Interestingly, the compound could target not only SARS-CoV-2 but also other coronaviruses that use similar RNA structures to promote frameshifting. These results suggest targeting PRF is a plausible, effective, and broad-spectrum antiviral strategy for SARS-CoV-2 and other coronaviruses.

**Fig. 1.**
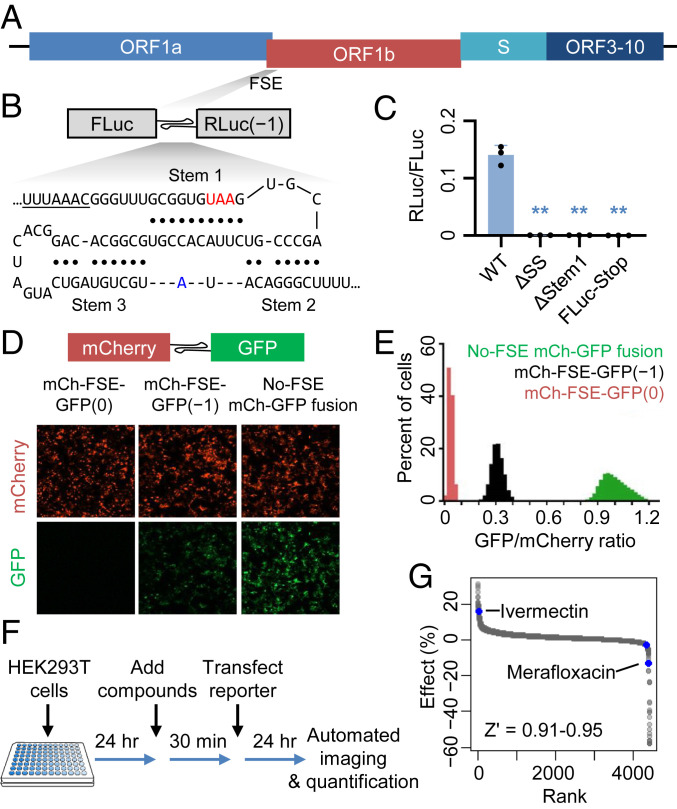
A high-throughput screen identifies SARS-CoV-2 PRF modulators. (*A*) Schematic illustration of the SARS-CoV-2 genome architecture, with the FSE indicated. (*B*) Schematic illustration of the dual luciferase-based −1 PRF reporter design. Watson−Crick base pairs are indicated by filled circles. Each of the three stems in the pseudoknot structure is labeled. The slippery sequence is underlined. The stop codon in the 0 frame is labeled in red. A13533, which varies from a cytosine in SARS-CoV, is labeled in blue. (*C*) Validation of the frameshift reporter. Mutating the slippery sequence (ΔSS), disrupting Stem 1 (ΔStem 1), or adding an in-frame stop codon upstream of pseudoknot eliminates frameshifting. **P < 0.01, two-sided *t* tests. (*D*) Representative images of cells transfected with mCherry-FSE_CoV-2_-GFP(0), mCherry-FSE_CoV-2_-GFP(−1), or mCherry-GFP. (*E*) Distributions of GFP/mCherry intensity ratios of individual cells transfected with mCherry-FSE_CoV-2_-GFP(−1) (black), mCherry-FSE_CoV-2_-GFP(0) (red), or mCherry-GFP (green). (*F*) Schematic illustration of high-throughput compound screen procedure. (*G*) Ranked effects of 4,434 tested compounds on mCherry-FSE_CoV-2_-GFP(−1) frameshift efficiency. Two validated active compounds, ivermectin and merafloxacin, are labeled in blue.

Severe acute respiratory syndrome coronavirus 2 (SARS-CoV-2), the etiological agent of COVID-19, belongs to a family of zoonotic human coronaviruses. Upon the entry of SARS-CoV-2 into host cells, the first set of viral proteins are translated from the long (>21-kb) open reading frame ORF1ab, which takes up approximately two-thirds of the viral genome (Fig. 1*A*) (1, 2). The ORF1ab-encoded polyprotein is subsequently processed into 16 individual nonstructural proteins (nsp) by two proteases, PLpro/nsp3 and 3CLpro/nsp5. The 3′ half of ORF1ab, ORF1b, encodes a variety of enzymes critical for viral transcription and replication, including an RNA-dependent RNA polymerase (RdRp/nsp12), an RNA helicase (Hel/nsp13), a proofreading exoribonuclease and N7-guanosine methyltransferase (ExoN/nsp14), an endonuclease (NendoU/nsp15), and a 2′-*O*-methyltransferase (nsp16). In all coronaviruses, translation of ORF1b requires a programmed −1 ribosomal frameshift (−1 PRF) (3). When ribosomes arrive at the end of ORF1a, instead of continuing elongation and soon terminating at an adjacent in-frame stop codon, a subset of ribosomes backtrack by one nucleotide and are repositioned in the −1 reading frame before continuing the translation elongation cycle, thereby producing a full-length ORF1ab polyprotein. A −1 PRF region often contains two main components: a heptanucleotide slippery sequence (UUUAAAC in SARS-CoV-2) and a downstream stable secondary structure acting as a frameshift-stimulatory element (FSE). The FSE secondary structures, most commonly being RNA pseudoknots (4, 5), are thought to facilitate −1 PRF in part by transiently pausing the incoming ribosome, allowing tRNAs to realign within the slippery sequence. In betacoronaviruses, including SARS-CoV-2, a three-stem pseudoknot (Fig. 1*B*) has been proposed to act as the productive conformation (3), although conformational dynamics of this region have recently been described (6–8).

In contrast to its wide adoption by RNA viruses, −1 PRF is much less prevalent in cellular mRNAs (5, 9, 10). Therefore, viral FSEs are attractive RNA targets for specific interference with viral gene expression (11, 12). Indeed, mutations and drugs that alter the efficiency of −1 PRF in HIV-1, which regulates Gag-Pol translation, have been shown to impede HIV-1 replication (11, 13, 14). In addition, an interferon-induced host protein, Shiftless (SHFL), has been shown to interact with HIV-1 FSE, inhibit −1 PRF, and restrict HIV-1 replication (15), suggesting that frameshift inhibition has become part of the host antiviral response. Several compounds have recently been shown to modulate −1 PRF of SARS-CoV-2 to varying degrees (16–18), although the specificity of these compounds and their antiviral activity remain unclear.

Here, we show that merafloxacin, a fluoroquinolone compound, specifically and robustly inhibits −1 PRF in betacoronaviruses including SARS-CoV-2. Inhibition of −1 PRF by merafloxacin impeded SARS-CoV-2 replication in Vero E6 cells, indicating that targeting −1 PRF represents a plausible antiviral strategy for SARS-CoV-2 and potentially other coronaviruses.

## Results

### A High-Throughput Screen Identifies −1 PRF Modulators for SARS-CoV-2.

To quantify −1 PRF efficiency in uninfected cells, we constructed a plasmid-based frameshifting reporter (Fig. 1*B*), replacing the stop codon of a firefly luciferase (FLuc) coding sequence (0 frame) with the SARS-CoV-2 FSE (nucleotides 13,460–13,548) including the slippery sequence and the three-stem pseudoknot, followed by a *Renilla* luciferase (RLuc) coding sequence in the −1 frame. Similar designs have been adopted by other recent studies (16, 19). Full-length reporter mRNA expression was confirmed by Northern blot analysis (*SI Appendix*, Fig. S1).

A −1-frame stop codon is embedded near the C terminus of FLuc, ruling out the possibility that RLuc translation initiated within FLuc coding sequence. To further confirm that the relative ratio between RLuc and FLuc activity indeed reported −1 PRF, we constructed and tested several negative controls: Deleting the slippery sequence (ΔSS), disrupting Stem 1 of the pseudoknot by a UAC trinucleotide deletion (ΔStem 1), and adding a 0-frame stop codon between FLuc and the slippery sequence (FLuc-Stop), all abolished RLuc activity (Fig. 1*C*), indicating that RLuc translation required the slippery sequence, the 3′ stimulatory structure, and upstream 0-frame translation, respectively. Based on a positive control construct in which FLuc and RLuc were translated continuously without frameshifting, we estimated the PRF efficiency to be ∼20% in HEK293T cells, consistent with previous reporter-based measurements (16, 20), although substantially lower than ribosome footprint profiling-based measurements (21) (*Discussion*).

To make the PRF reporter more suited for high-content screening, we replaced FLuc and RLuc with mCherry and GFP (Fig. 1*D*), respectively. Consistent with the luciferase-based reporter, mCherry-FSE-GFP(−1) reporter yielded reduced GFP signals compared to the in-frame mCherry-GFP fusion construct without an FSE (Fig. 1 *D* and *E*). Shifting GFP back to the 0 frame after FSE [mCherry-FSE-GFP(0)] completely abolished GFP signal (Fig. 1 *D* and *E*), consistent with the expectation that nonframeshifted ribosomes would terminate at an in-frame UAA stop codon within Stem 1 (Fig. 1*B*).

To identify chemical modifiers of −1 PRF, we treated HEK293T cells in 384-well plates with each of 4,434 compounds at 10 μM final concentration (Fig. 1*F* and Dataset S1*A*), which included 640 FDA-approved drugs, 1,600 compounds from the Pharmakon 1600 collection, and 1,872 compounds from a Tested-In-Human collection, and transfected cells with the mCherry-FSE-GFP(−1) reporter plasmids. Twenty-four hours after transfection, total cell numbers, as well as mCherry and GFP signals in transfected (mCherry^+^) cells were quantified. GFP/mCherry ratios were compared to the in-frame mCherry-GFP fusion positive control as well as the mCherry-FSE-GFP(0) negative control. Our high-content screening showed high robustness, with Z′ scores ranging between 0.91 and 0.95.

As expected, the vast majority of the tested compounds had little or no effect on the GFP/mCherry ratio (Fig. 1*G*). Screen actives were selected by using mean ± 3 SDs as cutoffs. False positives due to their intrinsic fluorescence (e.g., doxorubicin [red] and ampiroxicam [green]) were ruled out by manual inspection of images (Dataset S1*B*). We then repurchased the remaining eight candidates and tested each of them by using the luciferase-based PRF reporter assay. Among them, two compounds, ivermectin and merafloxacin, were validated as an enhancer and an inhibitor of −1 PRF, respectively (*SI Appendix*, Fig. S2). Although ivermectin has been recently shown to have anti–SARS-CoV-2 activity in vitro (22), it showed significant cytotoxicity in HeLa cells, as indicated by decreased ATP production (*SI Appendix*, Fig. S3*A*) and increased cell death (*SI Appendix*, Fig. S3*B*). In contrast, merafloxacin exhibited modest cytostatic effects at high concentrations (*SI Appendix*, Fig. S3*C*) and did not cause cell death (*SI Appendix*, Fig. S3*D*).

### Merafloxacin Specifically Inhibits −1 PRF of Betacoronaviruses.

**Fig. 2.**
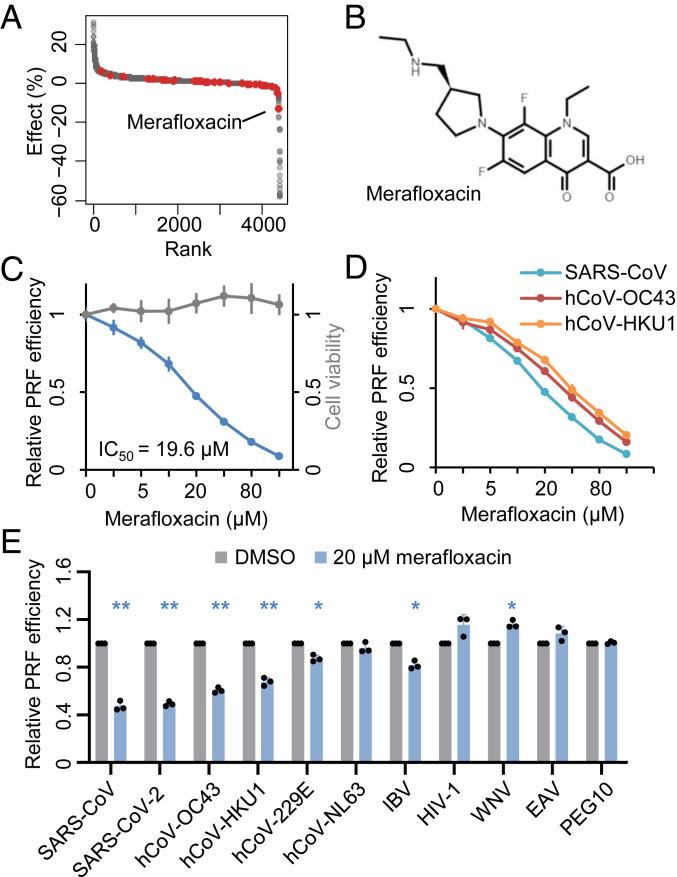
Merafloxacin specifically inhibits −1 PRF of betacoronaviruses. (*A*) Ranked effects of 4,434 tested compounds on mCherry-FSE_CoV-2_-GFP(−1) frameshifting efficiency. Fluoroquinolone compounds are labeled in red. (*B*) Chemical structure of merafloxacin. (*C*) Dose-dependent effects of merafloxacin on SARS-CoV-2 frameshifting reporter and HEK293T cell viability. (*D*) Dose-dependent inhibition of SARS-CoV, HCoV-OC43, and HCoV-HKU1 frameshifting by merafloxacin. (*E*) Effects of 20 µM merafloxacin on a panel of −1 PRF reporters. *P < 0.05; **P < 0.01, two-sided paired ratio *t* tests.

Merafloxacin, also known as CI-934, belongs to a large group of antibacterial compounds known as fluoroquinolones (23, 24). Interestingly, none of the 40 other fluoroquinolones in our compound library (Fig. 2*A*) nor additional fluoroquinolones subsequently tested (*SI Appendix*, Fig. S4) inhibited −1 PRF, suggesting that the varying moieties at N1, C7, and potentially other positions may be critical for frameshift inhibition (Fig. 2*B*). Merafloxacin robustly inhibited −1 PRF in SARS-CoV-2 in a dose-dependent manner, with an IC_50_ of ∼20 µM (Fig. 2*C*). By comparison, overexpression of SHFL, which has previously been shown to broadly inhibit −1 PRF (15), only reduced SARS-CoV-2 frameshifting by ∼25% (*SI Appendix*, Fig. S5), consistent with a recent report (25).

To test whether merafloxacin may inhibit other viral or cellular −1 PRF, we constructed additional reporters by using known FSEs from SARS-CoV, four common human coronaviruses (HCoV-HKU1, HCoV-OC43, HCoV-229E, and HCoV-NL63), avian infectious bronchitis virus (IBV), HIV-1, West Nile virus (WNV), equine arteritis virus (EAV), and human PEG10 (paternally expressed gene 10) mRNA. As expected from the nearly identical FSE sequences of SARS-CoV and SARS-CoV-2, which differ only by one unpaired nucleotide between Stem 2 and Stem 3 (C13533A), merafloxacin inhibited −1 PRF of both coronaviruses with virtually equal efficacy (IC_50_ = 20 µM) (Fig. 2*D*). Interestingly, merafloxacin showed similar activity against −1 PRF of two other human betacoronaviruses, HCoV-HKU1 (IC_50_ = 30 µM) and HCoV-OC43 (IC_50_ = 39 µM) (Fig. 2*D*). In contrast, merafloxacin had much weaker activity against −1 PRF of alphacoronaviruses, HCoV-229E and HCoV-NL63 (Fig. 2*E*), the FSEs of which form an elaborate pseudoknot structure that substantially differs from those of betacoronaviruses (26). The two-stem pseudoknot FSE of IBV (20), a gammacoronavirus, was also largely insensitive to merafloxacin (Fig. 2*E*). Last, merafloxacin did not inhibit −1 PRF of HIV-1, WNV, EAV, nor human PEG10 mRNA (Fig. 2*E*). These results indicate that merafloxacin specifically targets betacoronavirus FSEs, which share a common three-stem pseudoknot architecture.

Merafloxacin did not affect the translation of upstream FLuc translation (*SI Appendix*, Fig. S6*A*), global translation (*SI Appendix*, Fig. S6*B*), nor ribosome association of the reporter mRNA in HEK293T cells (*SI Appendix*, Fig. S6*C*). To further rule out the possibility that the different amino acid sequences in each reporter may influence the effect of merafloxacin, we inserted 2A “StopGo” peptides from porcine teschovirus-1 (P2A) both upstream and downstream of each FSE (*SI Appendix*, Fig. S7*A*). Full-length reporter mRNA expression was confirmed by Northern blot analysis (*SI Appendix*, Fig. S7*B*). Importantly, we observed similar dose-dependent inhibition of SARS-CoV-2 frameshifting by merafloxacin (*SI Appendix*, Fig. S7*C*) as well as similar selectivity toward betacoronaviruses (*SI Appendix*, Fig. S7*D*). In addition, merafloxacin did not affect reporter mRNA abundance (*SI Appendix*, Fig. S7*E*) nor cell viability (*SI Appendix*, Fig. S7*F*). Furthermore, merafloxacin inhibited −1 PRF of in vitro-transcribed SARS-CoV-2 reporter mRNAs (*SI Appendix*, Fig. S7*G*), thereby ruling out any potential artifact from nuclear expression of the reporter mRNAs.

### Frameshift Inhibition by Merafloxacin Is Robust to Mutations within FSE.

**Fig. 3.**
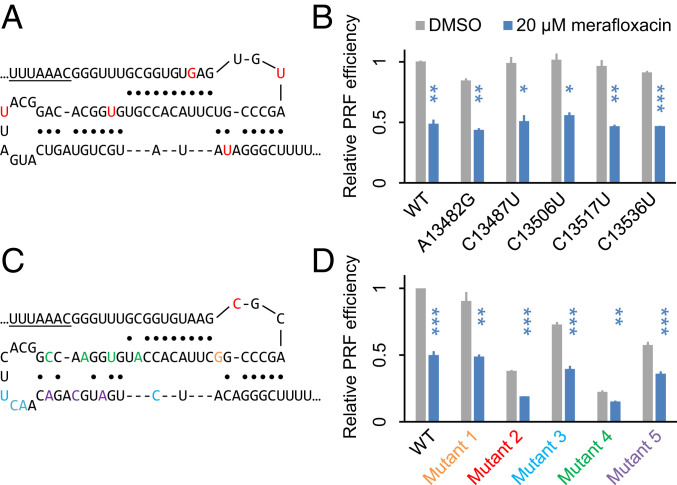
Frameshift inhibition by merafloxacin is robust to mutations within FSE. (*A*) Positions of the naturally occurring C-to-U mutations, labeled in blue. (*B*) Effects of merafloxacin on −1 PRF of each FSE natural variant. ***P < 0.001, two-sided ratio *t* tests. (*C*) Positions of the structure-perturbing, synonymous mutations. Each group of mutations is labeled in the same color. (*D*) Effects of merafloxacin on −1 PRF of each FSE mutant. *P < 0.05; **P < 0.01; ***P < 0.001, two-sided paired ratio *t* tests.

Rapidly replicating viruses constantly acquire and accumulate nondeleterious mutations. To test whether mutations within FSE may confer resistance to merafloxacin, we first introduced mutations that have been documented in the current SARS-CoV-2 genome sequence database (27). Consistent with the essential role of −1 PRF, mutations within FSE are exceedingly rare. Out of five recurrent single-nucleotide substitutions that have been observed in two or more samples (27), only two have alternative allele frequencies >0.1% (0.16% for C13487U, 5.5% for C13536U) (Fig. 3*A*). A13482G changes an A:U to a G:U pair in Stem 1. C13506U and C13536U each changes a C:G to a U:G pair in Stem 3 and Stem 2, respectively. C13487U and C13517U are in the terminal loops of Stem 2 and Stem 3, respectively. Therefore, all the recurrent mutations preserve the three-stem pseudoknot architecture. Regardless of the baseline effects of these mutations, merafloxacin inhibited −1 PRF in all variants with similar efficacy (Fig. 3*B*).

Having tested these structure-preserving natural mutations, we next introduced sets of synonymous mutations intended to perturb the pseudoknot structure (Fig. 3*C*). U13494G (mutant 1, orange), which disrupts a basal U:A pair in Stem 2, only slightly reduced frameshift efficiency (Fig. 3*D*), consistent with a recently reported cryo-EM structure in which G13493 and U13494 are unpaired (19). Also consistent with this cryo-EM structure, in which Loop 1 forms multiple contacts with ribosomal proteins (19), is a 62% reduction in frameshift efficiency caused by U13485C (mutant 2, red) (Fig. 3*D*). Indeed, both U13485 and the subsequent G13486 are invariable among all human coronaviruses (28). A13519U;G13520C;U13521A;A13533C (mutant 3, cyan) disrupts the palindromic sequence in the terminal loop of Stem 3 (29), causing a 27% decrease in frameshifting. As expected from the crucial role of Stem 1, G13503A;C13506U;C13509A;A13512C (mutant 4, green), which disrupt multiple base pairs in Stems 1 and 3, caused the largest (77%) reduction in frameshift efficiency. Last, U13524A;U13527C;C13530A (mutant 5, purple) disrupts Stem 3, causing a 42% decrease in frameshifting (Fig. 3*D*). It should be noted that in addition to structural perturbation, these synonymous mutations may have additional effects such as influencing translation speed by changing codon optimality. Nevertheless, despite the wide range of effects of these mutations on the baseline −1 PRF efficiency, merafloxacin significantly inhibited frameshifting in all variants (Fig. 3*D*), with only a slightly diminished effect (33% inhibition versus 50% in WT) on the most severely crippled mutant 4 (Fig. 3*D*). These results suggest that −1 PRF inhibition of merafloxacin is highly robust to perturbations to the FSE sequence or structure.

### Merafloxacin Impedes SARS-CoV-2 Replication.

**Fig. 4.**
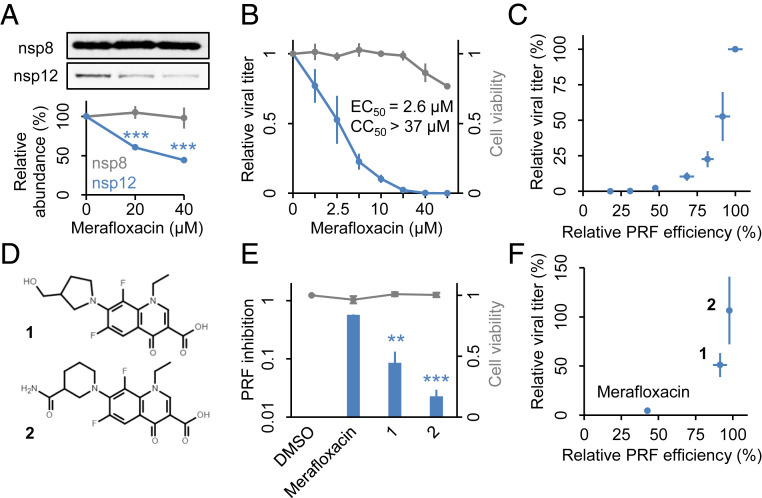
Merafloxacin impedes SARS-CoV-2 replication in Vero E6 cells. (*A*) Relative abundance of nsp8 and nsp12 in DMSO- or merafloxacin-treated Vero E6 cells 48 h after SARS-CoV-2 infection (MOI = 0.05). (*B*) Effects of merafloxacin on relative viral growth and Vero E6 cell viability. EC_50_ and CC_50_ concentrations are shown. (*C*) Relationship between viral titers and relative PRF efficiency at each merafloxacin concentration, using data from *A* and Fig. 2*C*. (*D*) Chemical structures of two merafloxacin analogs with modified C7 moieties. (*E*) Effects of merafloxacin analogs on SARS-CoV-2 frameshifting reporters and Vero E6 cell viability. (*F*) Relationship between viral titers and relative PRF efficiency for each compound. **P < 0.01; ***P < 0.001, two-sided paired ratio *t* tests.

Having observed its anti-frameshifting activity in PRF reporter assays, we went on to test whether merafloxacin could impede −1 PRF and viral growth in SARS-CoV-2–infected cells. After infecting Vero E6 cells with SARS-CoV-2, we treated cells with varying concentrations of merafloxacin and quantified the abundance of nsp8 and nsp12 encoded by ORF1a and ORF1b, respectively. As expected, the relative abundance between nsp12 and nsp8 was substantially reduced by merafloxacin (Fig. 4*A*). Concomitantly with frameshift inhibition, merafloxacin impeded SARS-CoV-2 replication, with an EC_50_ of 2.6 μM and an EC_90_ of 12 μM, without causing substantial cytotoxicity (Fig. 4*B*). Correlating viral titer measurements with the effect of merafloxacin on −1 PRF efficiency revealed a near-exponential decrease in virus yield as −1 PRF was increasingly inhibited (Fig. 4*C*), suggesting that −1 PRF efficiency is rate-limiting for SARS-CoV-2 replication. Consistent with its targeting specificity toward betacoronavirus FSEs (Fig. 2*E*), merafloxacin showed greater antiviral activity for HCoV-OC43 than for HCoV-229E (*SI Appendix*, Fig. S8). Also consistent with its lack of anti-frameshifting activity on HIV-1 FSE (Fig. 2*E*), merafloxacin showed no antiviral activity against an HIV-1 reporter virus in Jurkat T cells (*SI Appendix*, Fig. S9).

To further assess the relationship between frameshifting inhibition and antiviral activity of merafloxacin, we designed and synthesized merafloxacin analogs with altered moieties at C7, which distinguished merafloxacin from other fluoroquinolones (Fig. 4*D*). As expected, shortening the distal sidechain while keeping the pyrrolidine (compound **1**) reduced the anti-frameshifting activity (Fig. 4*E*), whereas replacing the pyrrolidine with a piperidine (compound **2**) almost completely abolished the anti-frameshifting activity. Concomitant with the changes in frameshift inhibition, the antiviral activity of these analogs was also reduced or abolished, respectively (Fig. 4*F*), suggesting that the anti-frameshifting and antiviral activities of merafloxacin are tightly coupled.

## Discussion

With the promptly made available SARS-CoV-2 genome sequence (30) as well as the prior knowledge of common features of viral FSEs (5), especially those of coronaviruses (3, 20), we and others (16) have been able to model −1 PRF of SARS-CoV-2 using similar bicistronic reporters in heterologous systems. The versatility of these reporters allowed us to perform large-scale compound screens to identify and validate chemical modifiers of this crucial aspect in SARS-CoV-2 gene expression. While these heterologous systems can capture the essential events during −1 PRF, they often do not fully recapitulate −1 PRF in the native viral genomic RNA context. Indeed, while our measurement of the baseline SARS-CoV-2 frameshifting efficiency (15–20%) is in line with other reporter-based studies (16, 20), it is lower than those based on ribosomal occupancy (30–50%) (2). The apparent discrepancy of these two types of measurements may be attributed to a combination of factors. On one hand, the FSE RNA taken out of its native genome context may lose certain interactions with its native flanking sequence (6, 8, 31) and/or gain interactions with the artificial reporter sequences, which collectively affect the fraction of reporter RNAs folded into the native, productive conformation. Furthermore, −1 PRF in the native context may be regulated by additional viral and/or host proteins, which would be missed by heterologous reporter systems. On the other hand, ribosome footprint profiling could overestimate or underestimate frameshifting efficiency due to a variety of factors, such as RNA fragments protected by secondary structures, nonribosomal proteins, or paused ribosomes. Future studies that can systematically eliminate each confounding factor will presumably resolve the apparent discrepancy. Nevertheless, the effects of merafloxacin on both nsp12 abundance and SARS-CoV-2 replication strongly suggest that findings from our heterologous reporter system indeed translate to −1 PRF during SARS-CoV-2 infection.

A variety of RNA viruses have been shown to be exquisitely sensitive to subtle changes in the expression of replication machineries, which are finely tuned holoenzyme complexes. For example, SARS-CoV replication is severely impacted by mutations that modestly reduce frameshifting efficiency (32). Similarly, mutations that increase or decrease frameshifting efficiency by approximately twofold profoundly inhibit replication of LA virus, a dsRNA virus of yeast (33). The replication of hepatitis C virus, another positive-strand RNA virus, is highly sensitive to a mutation that subtly improves the kinetics of NS4B-5A polyprotein cleavage (34). Furthermore, rearranging the order of genes within vesicular stomatitis virus, a negative-strand RNA virus, alters the ratio of replication proteins by approximately twofold yet causes 2- to 4-log reductions in viral replication and strong attenuates virulence in animals (35). The identification of merafloxacin as a betacoronavirus −1 PRF inhibitor allowed us to further evaluate the degree to which replication of SARS-CoV-2, which has a highly efficiency FSE, may be similarly sensitive to small changes in −1 PRF efficiency. Hypothetically, high −1 PRF efficiency could allow SARS-CoV-2 to tolerate a small decrease in PRF efficiency and still produce enough replicase components encoded by ORF1b. In this scenario, a large reduction in −1 PRF would be required to inhibit viral growth, and drugs targeting −1 PRF would be less effective for SARS-CoV-2 than for viruses with lower −1 PRF efficiency (e.g., HIV-1). Our results strongly argued against this possibility. Instead, the observed near-exponential relationship between viral titer and frameshifting efficiency (Fig. 4*C*) points to a simple model in which ORF1b translation is a rate-limiting step for SARS-CoV-2 replication, thereby providing strong support for targeting −1 PRF as an effective antiviral strategy for SARS-CoV-2 and possibly other RNA viruses with high frameshifting efficiency.

Other nonexclusive models might also explain the high sensitivity of SARS-CoV-2 replication to −1 PRF inhibition. For instance, coronaviruses may require high PRF efficiency to achieve an optimal stoichiometry between the components of its replicase–transcriptase complex, and the optimum may be disrupted by even a small reduction in −1 PRF efficiency. In this case, increasing PRF efficiency might also be detrimental to the virus, as has been previously shown for LA virus (33) and HIV-1 (14). Furthermore, multiple ORF1b-encoded replicase–transcriptase components may be rate-limiting for replication, in which case small reductions in each of them would multiply and collectively cause a larger effect. Last, our results do not rule out additional, frameshifting-independent actions of merafloxacin, which may also contribute to its antiviral activity.

The molecular mechanism by which merafloxacin inhibits −1 PRF is currently unknown. The simplest model would involve the direct binding between merafloxacin and the FSE RNA. Such an interaction may either destabilize the pseudoknot conformation, thereby reducing ribosome pausing and subsequent frameshifting or, on the contrary, further stabilize the pseudoknot structure, thereby causing prolonged stalling, collisions, and/or queuing of the incoming ribosomes. Contrasting ribosome occupancy profiles of either the −1 PRF reporter RNA or the viral genomic RNA with and without merafloxacin treatment may distinguish between these scenarios.

A second possibility is that merafloxacin might stabilize an alternative and unproductive (i.e., non-frameshift-stimulating) FSE conformation. At least one alternative structure with two nested stems has been shown to form in SARS-CoV-2–infected cells by a recent study using dimethyl sulfate probing (6). Notably, our ΔStem1 mutant is fully compatible with this alternative conformation, yet it lost all frameshift-stimulating activity (Fig. 1*C*), consistent with the notion that this stem-loop structure is an unproductive conformation. A separate study using in-cell SHAPE probing did not detect this stem-loop structure, but instead observed substantial conformational flexibility of Stem 3 with a 20% folding probability (8). Therefore, merafloxacin could plausibly interact and stabilize one or more of the alternative structures, thereby decreasing the fraction of RNAs adopting the productive FSE conformation.

Finally, merafloxacin might target one or more host factors that mediate or modulate −1 PRF. Although such factors have not been systematically identified, cellular RNA helicases would presumably help unfold the pseudoknot after the ribosome has shifted to the −1 frame and before it continues to translate ORF1b. Considering that the known targets of fluoroquinolones are bacterial DNA topoisomerases (24), it will be interesting to determine whether their metazoan analogs, some of which have been shown to act on RNAs (36), may be targeted by merafloxacin.

## Methods

### Dual-Luciferase PRF Reporter Assay.

HeLa and HEK293T cells were cultured in DMEM with 10% fetal bovine serum (Thermo Fisher). PRF reporter plasmid DNAs were transfected using Lipofectamine 2000 (Thermo Fisher) according to the manufacturers’ instructions. Twenty-four hours after transfection, cells were washed once with phosphate-buffered saline (PBS), and lysed in Glo Lysis Buffer (Promega) at room temperature for 5 min. One microliter of lysate was diluted with 39 µL of PBS before being mixed with 40 µL of Dual-Glo FLuc substrate (Promega). After 10 min, FLuc activity was measured in a GloMax 20/20 luminometer (Promega). Subsequently, 40 µL of Dual-Glo Stop and Glo reagent was added to the mixture, incubated for 10 min, and measured for RLuc luminescence. The ratio between RLuc and FLuc activities was calculated as frameshift efficiency.

### High-Throughput Compound Screen.

HEK293T cells were plated in 384-well plates at the density of 5,000 cells/well. The next day, screened compounds (20 nL of 10 mM stock in DMSO) were added to 20 µL of cells using ECHO acoustic dispenser (Labcyte), resulting in 10 mM compound and 0.1% DMSO final concentrations. Cells were treated with candidate compounds for 30 min before transfection of 15 ng of mCherry-FSE-GFP(−1) plasmid DNA in each well. Twenty-four hours after transfection, cell nuclei are stained with Hoechst dye. Cell nuclei, mCherry, and GFP signals are imaged using an automated fluorescent microscope (InCell 2200; GE) with a 20× objective, and the acquired images are quantified using the CellProfiler image analysis package. Cell nuclei numbers were quantified as the metric for cell viability. Mean RFP intensity values were first measured in all cells to identify transfected (mCherry-positive) cells, and GFP/mCherry mean intensity ratio was quantified in transfected cells as the metric for PRF efficiency. The no-FSE mCherry-GFP fusion plasmid and the mCherry-FSE-GFP(0) plasmid were used as positive and negative controls for elevated and reduced GFP expression levels, respectively. Screen actives were selected using mean ± 3 SDs as cutoffs.

A total of 4,434 compounds were screened (Dataset S1), including 640 compounds from the FDA-approved library (ENZO), 1,600 compounds from Pharmakon collection (Microsource), and 1,872 compounds from the Tested-In-Humans collection (Yale Center for Molecular Discovery). Additional quinolone compounds were either purchased from Cayman Chemical or synthesized by New England Discovery Partners.

### Northern Blotting.

RNA probe complementary to a fragment (∼350 nt) of RLuc sequence was in vitro transcribed by using a HiScribe T7 high-yield RNA synthesis kit (NEB) with DIG-11-UTP (Roche) and 200 ng of template DNA (PCR product), and purified by using Monarch RNA Cleanup Kit (NEB). Total RNA was extracted from HEK293T cells using TRIzol (Invitrogen) and treated with TurboDNase at 37 °C for 30 min. For each sample, 5 µg of total RNA was mixed with 5× RNA loading buffer (2.5 mg/mL bromophenol blue, 12 mM EDTA, 2.76% formaldehyde, 20% glycerol, 30.84% formamide, 80 mM MOPS, 20 mM NaOAc), denatured at 65 °C for 10 min, rapidly cooled on ice, and loaded on a 1% formaldehyde agarose gel (1% agarose, 0.67% formaldehyde, 20 mM MOPS, 5 mM NaAc, 2 mM EDTA, pH 7.0). Formaldehyde agarose gels were run at 5 V/cm in 1× running buffer (0.74% formaldehyde, 20 mM MOPS, 5 mM NaOAc, 2 mM EDTA, pH 7.0), stained with SYBR Gold (Thermo Fisher) in 1× TBE buffer, and imaged using a Gel Doc XR system (Bio-Rad). RNA was transferred to a BrightStar-Plus positively charged nylon membrane (Thermo Fisher) by overnight capillary transfer in 20× SSC and cross-linked by 254-nm UV. After prehybridization, membranes were incubated with 100 ng/mL denatured DIG-labeled RNA probe in DIG EasyHyb buffer (Roche) at 68 °C overnight, washed twice in 2× SSC, 0.1% SDS at room temperature, twice in 0.1× SSC, 0.1% SDS at 68 °C, and incubated with anti-DIG-alkaline phosphatase conjugate (Roche). Chemiluminescent signals were developed using CDP-Star (Roche) according to the manufacturer’s instruction and detected by using an Odyssey CLx system (Li-Cor).

### Western Blotting.

Transfected HEK293T cells or SARS-CoV-2–infected Vero E6 cells were lysed in RIPA buffer on ice for 10 min. After 10-min centrifugation at 4 °C, 20,000 × *g*, whole-cell lysates were mixed with 4× LDS sample buffer (Invitrogen) and denatured at 95 °C for 5 min. Samples were loaded on a 4–12% Bis-Tris SDS-PAGE gel, run at 200 V for 45 min in MOPS buffer, and transferred onto a nitrocellulose membrane (Bio-Rad) in an XCell II Blot module (Invitrogen) (15 V, 45 min). After 1-h blocking with 5% nonfat dry milk in PBST, the membrane was incubated with primary antibodies (rabbit anti-C19ORF66, Invitrogen, #PA5-59815; mouse anti-β-Actin, Cell Signaling, #3700; rabbit anti-nsp8, Novus Biologicals, #NBP2-89180; rabbit anti-nsp12, ProSci Inc., #9267) diluted (1:2,000) in 5% milk/PBST at 4 °C with slow shaking overnight. After incubation, membranes were rinsed three times with PBST, and incubated with IR680- or IR800-conjugated secondary antibodies (Li-Cor) diluted (1:10,000) in 5% milk/PBST at room temperature for 1 h. After three rinses with PBST, membranes were imaged using an Odyssey CLx system (Li-Cor).

### Viral Plaque Formation Assay.

Plaque formation assays for SARS-CoV-2, HCoV-229E, and HCoV-OC43 were performed in Vero E6, HuH-7.5, and MA104 cells (ATCC), respectively. Briefly, cells were seeded at 2–4 × 10^5^ cells/well in 12-well plates. The following day, cells were incubated with viral stock (multiplicity of infection [MOI] = 0.05) for 1 h and washed twice with PBS. Merafloxacin diluted in DMSO was added to the media, mixed briefly, and incubated at 37 °C. After 2 d, media were collected, centrifuged, and supernatants were stored at −80 °C. To quantify viral titers, the collected media were first serially diluted 10-fold with fresh media. Two hundred microliters of each dilution were added to near-confluent cells in six-well plates and incubated at 37 °C for 1 h with gentle rocking. Subsequently, overlay media (DMEM, 2% FBS, 0.6% Avicel RC-581) was added to each well. After 3 d, cells were fixed with 10% formaldehyde for 30 min, stained with crystal violet for 30 min, and rinsed with deionized water to visualize plaques.

### HIV-1 Antiviral Assay.

Jurkat T cells were infected with a replication-competent HIV-1 virus (NL4-3-dNef-GFP) by spinoculation. Tested compounds were added 2 h after infection. DMSO and an HIV-1 reverse transcriptase inhibitor tenofovir (10 μM) were used as negative and positive controls, respectively. Three days after infection, cell viability (fixable near-IR dead cell staining; Thermo Fisher) and HIV-1 infection were measured as percent GFP positive cells out of viable cells using flow cytometry.

## Supplementary Material

Supplementary File

Supplementary File

## Data Availability

All study data are included in the article and/or supporting information.

## Note

During the review of our manuscript, confirmatory results on the antiframeshifting and antiviral activities of merafloxacin have been reported by Bhatt *et al*. (19).

## Change History

July 9, 2021: The Acknowledgments have been updated.
