# Supplementary material for: Restriction of SARS-CoV-2 replication by targeting programmed −1 ribosomal frameshifting
Source: Proc Natl Acad Sci U S A. 2021 Jun 14;118(26):e2023051118. doi: 10.1073/pnas.2023051118 (PMC8256030; doi:10.1073/pnas.2023051118)
Supplement: Supplementary File [file pnas.2023051118.sapp.pdf]

PNAS
www.pnas.org

Supplementary Information for

Restriction of SARS-CoV-2 Replication by Targeting Programmed −1 Ribosomal Frameshifting

Yu Sun, Laura Abriola, Rachel O. Niederer, Savannah F. Pedersen, Mia M. Alfajaro, Valter Silva Monteiro, Craig B. Wilen, Ya-Chi Ho, Wendy V. Gilbert, Yulia V. Surovtseva, Brett D. Lindenbach, Junjie U. Guo

Brett D. Lindenbach
Email: brett.lindenbach@yale.edu

Junjie U. Guo
Email: junjie.guo@yale.edu

This PDF file includes:

Figures S1 to S9

Other supplementary materials for this manuscript include the following:

Datasets S1

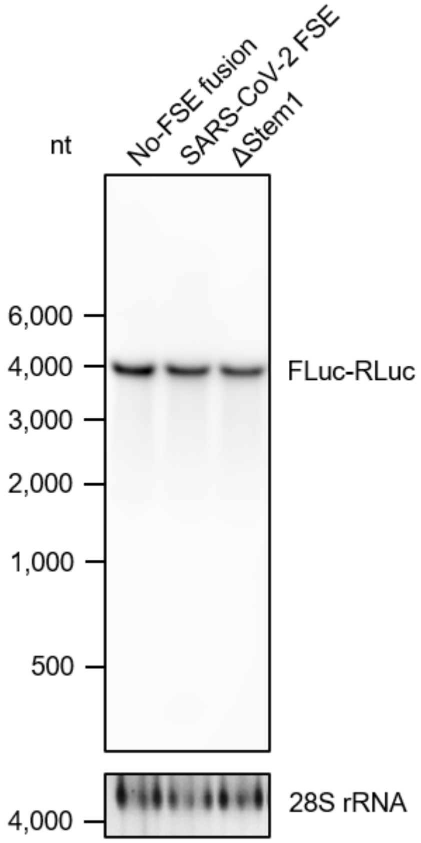

Fig. S1. Expression of −1 PRF reporter mRNA.

Northern blotting with an anti-RLuc probe showed the expression of a single predominant transcript with an expected length of ~4kb. 28S rRNA is shown as loading control.

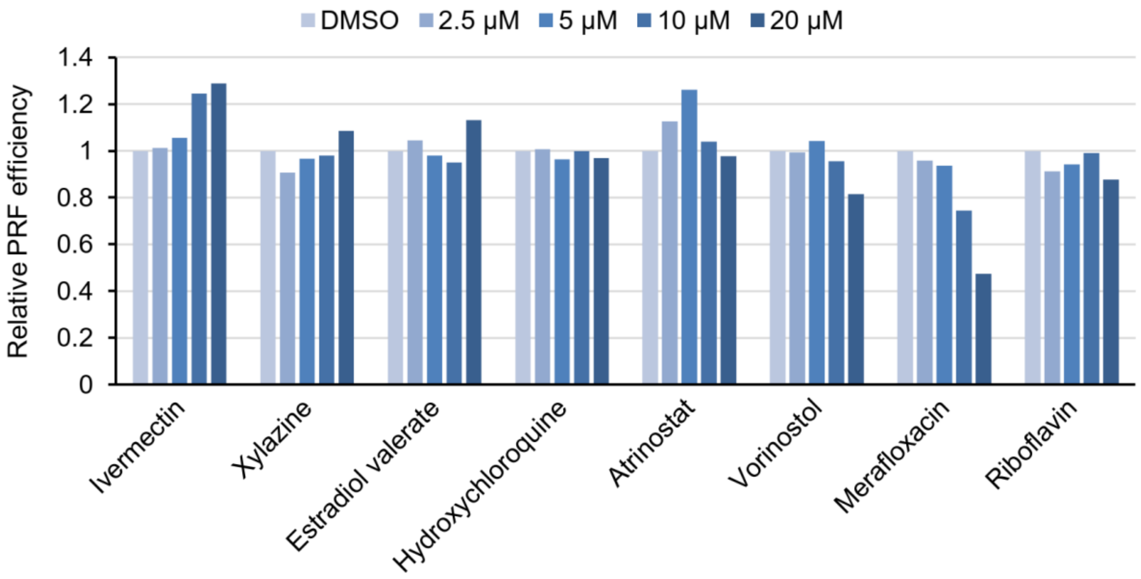

Fig. S2. Secondary screen of candidate compounds.

Candidate compounds from the microscopy screen were tested using the dual luciferase-based −1 PRF reporter assays.

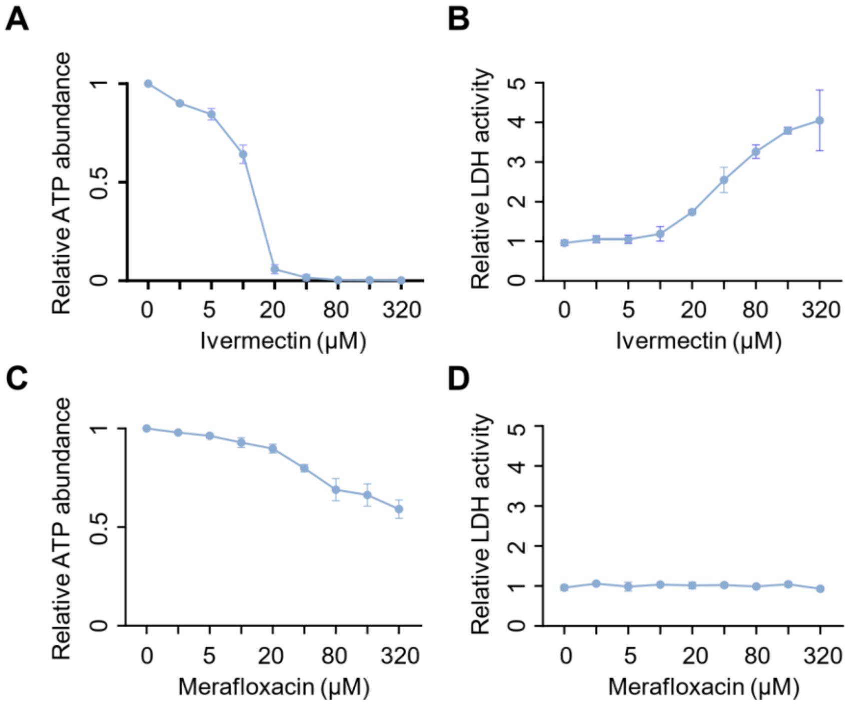

Fig. S3. Effects of ivermectin and merafloxacin on cell viability.

Cytotoxicity of ivermectin (**A, B**) and merafloxacin (**C, D**) in HeLa cells was quantified by ATP production (**A, C**) and LDH release (**B, D**).

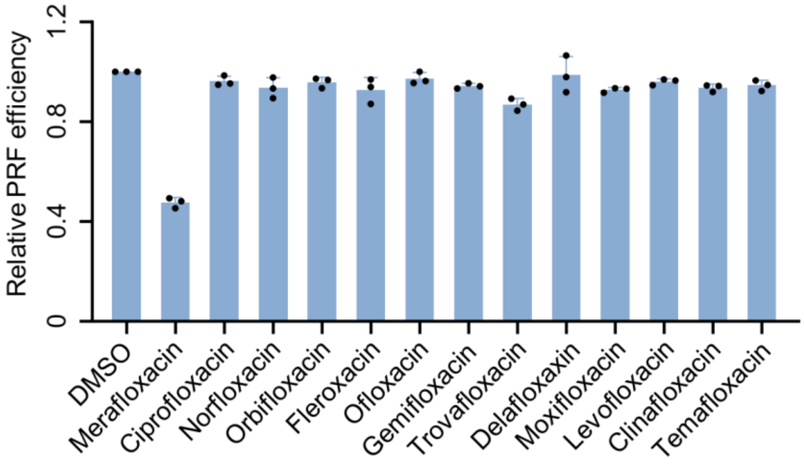

Fig. S4. Effect of additional fluoroquinolone compounds on −1 PRF.

Each compound was added to a final concentration of 20 μM. Dual luciferase-based −1 PRF reporter assays were used to quantify the effect of each compound.

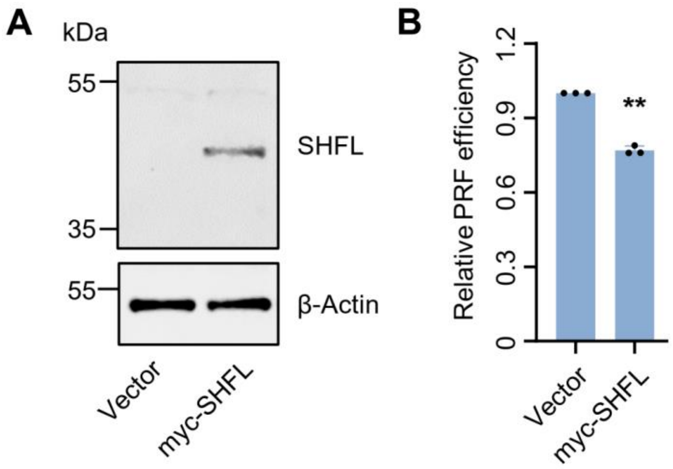

Fig. S5. Effect of SHFL overexpression on −1 PRF.

(**A**) Western blot showing the overexpression of myc-tagged SHFL in HEK293T cells.
(**B**) Effect of myc-SHFL overexpression on −1 PRF efficiency measured with luciferase assays.

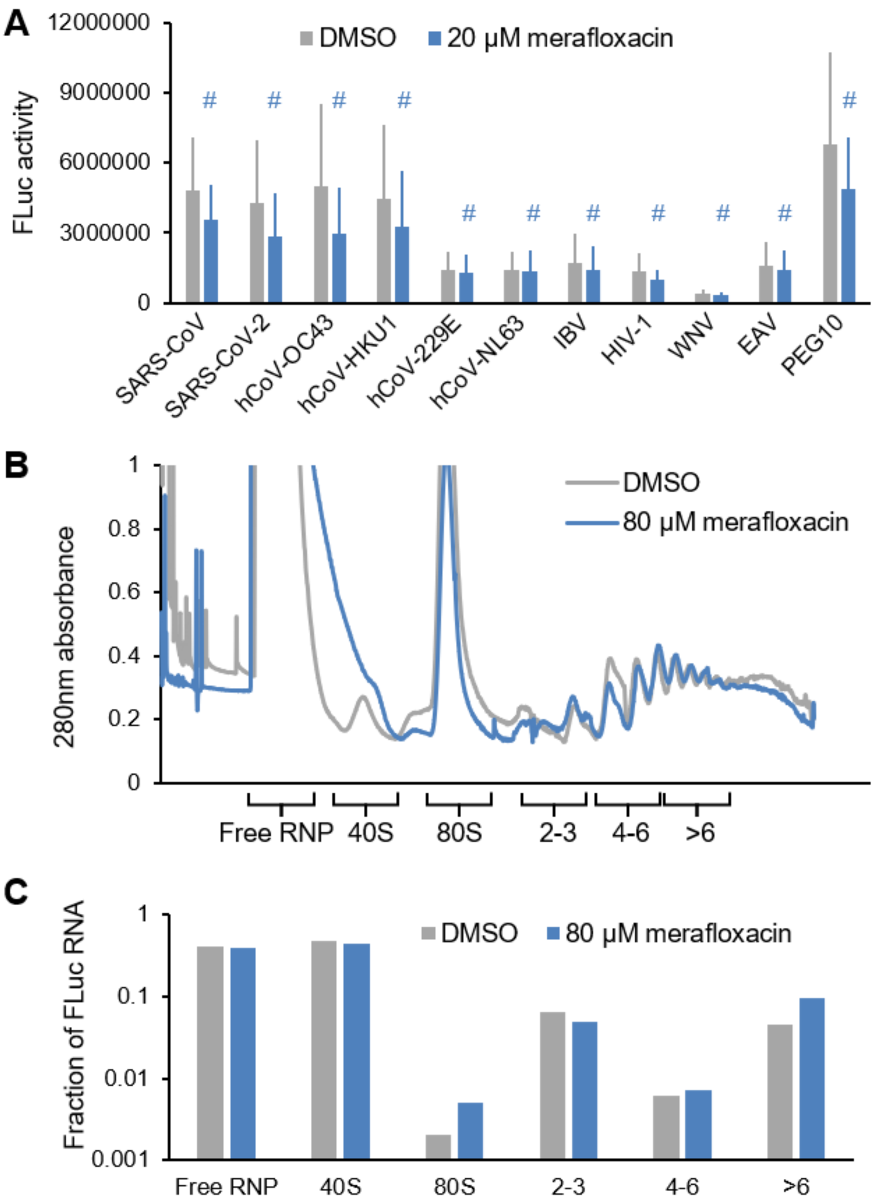

Fig. S6. Effects of merafloxacin on global translation and ribosome association of reporter RNA.

(**A**) FLuc activity of −1 PRF reporters with and without 20 μM merafloxacin. #, $p>0.05$, two-tailed paired Student's t test.
(**B**) Polysome profiles of HEK293 cells treated with DMSO or 80 μM merafloxacin for 24 hours. Fractions collected for RT-qPCR are indicated.
(**C**) Relative distribution of reporter mRNA in each fraction with and without 80 μM merafloxacin.

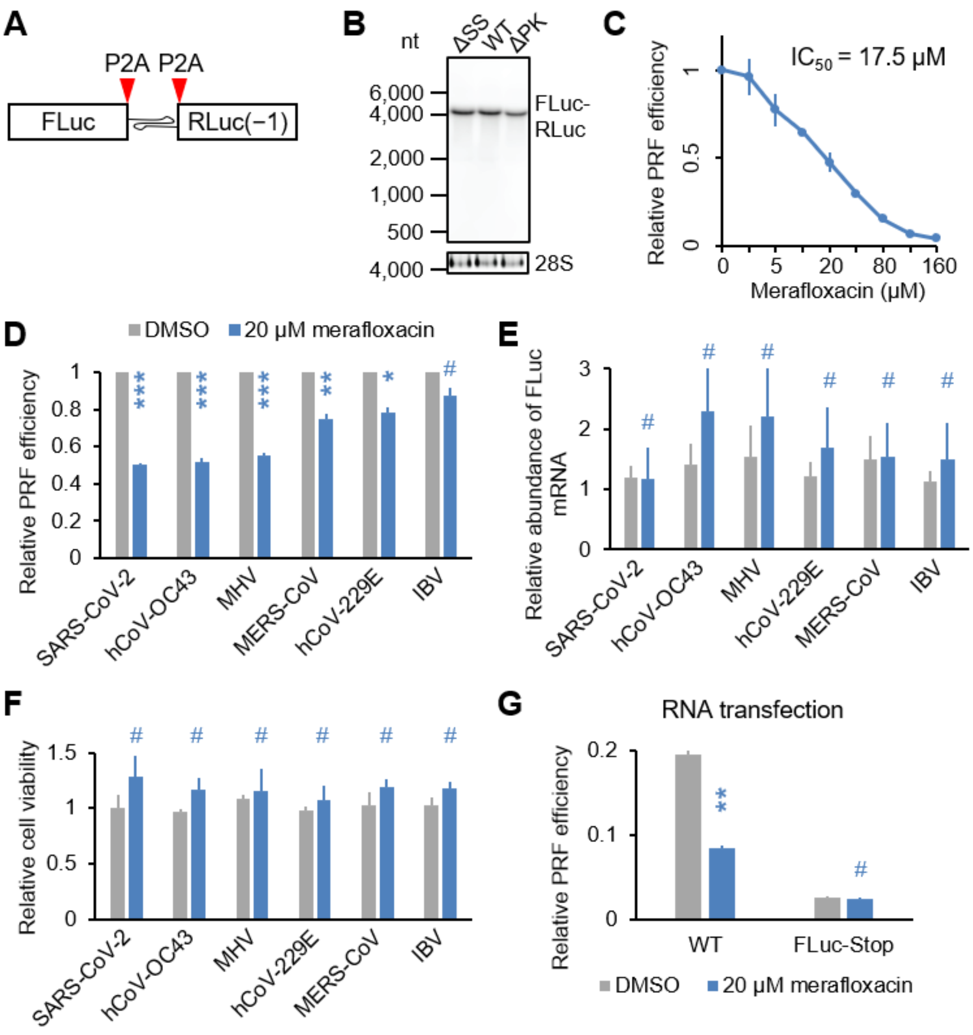

Fig. S7. Validation of anti-frameshifting activity with an improved −1 PRF reporter.

(**A**) Schematic illustration of a −1 PRF reporter with P2A peptide sequences flanking the FSE.
(**B**) Northern blot showing the expression of a single transcript with an expected length of ~4kb. ΔSS, in-frame positive control with a disrupted slippery sequence. ΔPK, negative control with the pseudoknot region deleted. 28S rRNA serves as loading control.
(**C**) Dose-dependent inhibition of the improved SARS-CoV-2 PRF reporter by merafloxacin. IC_{50} concentration is shown.
(**D**) Effect of merafloxacin on −1 PRF efficiency measured with the improved −1 PRF reporters.
(**E**) Effect of merafloxacin on the FLuc mRNA abundance.
(**F**) Effect of merafloxacin on HEK293T cell viability.
(**G**) Effect of merafloxacin on −1 PRF efficiency of in vitro transcribed RNAs with either wild-type (WT) SARS-CoV-2 FSE or an in-frame stop codon inserted upstream of FSE (FLuc-Stop). #, *p*>0.05; *, *p*<0.05; **, *p*<0.01; ***, *p*<0.001, two-tailed Student's t tests.

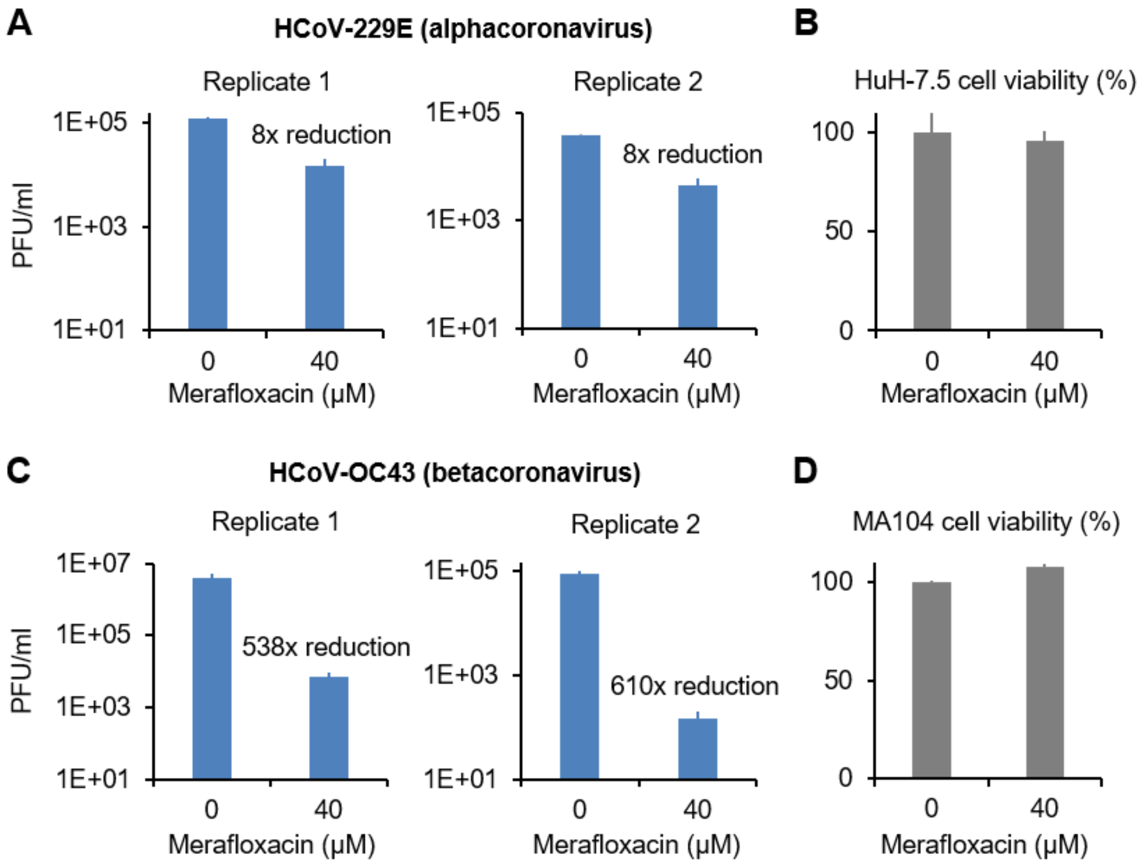

Fig. S8. Antiviral activity of merafloxacin on HCoV-229E and HCoV-OC43.

(**A**) Antiviral activity of merafloxacin against HCoV-229E in HuH-7.5 cells. Fold reductions are shown.
(**B**) Effect of merafloxacin on HuH-7.5 cell viability.
(**C**) Antiviral activity of merafloxacin against HCoV-OC43 in MA104 cells. Fold reductions are shown.
(**D**) Effect of merafloxacin on MA104 cell viability.

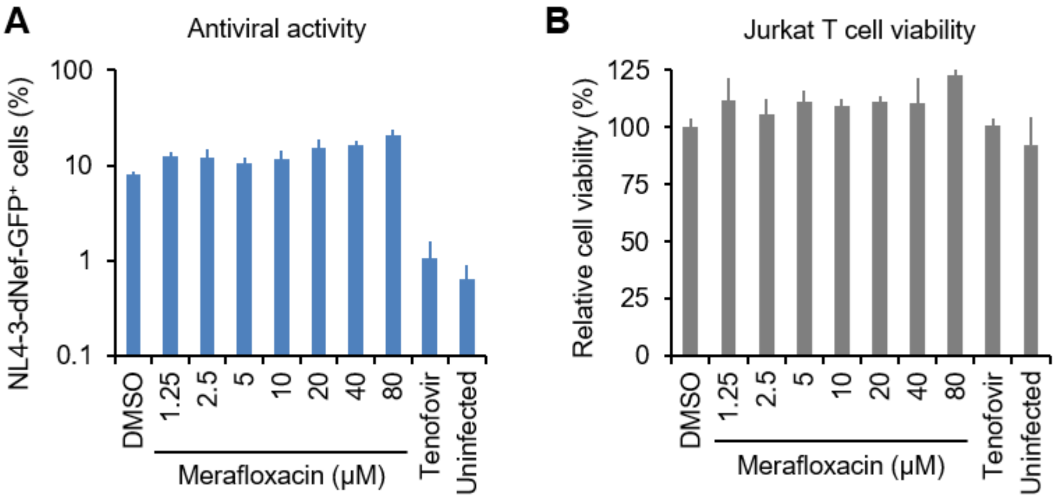

Fig. S9. Antiviral activity of merafloxacin on HIV-1.

(**A**) Antiviral activity of merafloxacin and control compounds against HIV-1 in Jurkat T cells.
(**B**) Effects of merafloxacin and control compounds on Jurkat T cell viability, normalized to DMSO.

Dataset S1 (separate file). High-throughput screening results.
